# Improving Multi-Agent Generative Adversarial Nets with Variational Latent Representation

**DOI:** 10.3390/e22091055

**Published:** 2020-09-21

**Authors:** Huan Zhao, Tingting Li, Yufeng Xiao, Yu Wang

**Affiliations:** School of Information Science and Engineering, Hunan University, Changsha 410082, China; tingting1225@hnu.edu.cn (T.L.); hnxiaoyf@hnu.edu.cn (Y.X.); yuwang18@hnu.edu.cn (Y.W.)

**Keywords:** diversity, generative adversarial networks, mode collapsing, multi-agent generator, quality, variable auto-encoder, variational latent representations

## Abstract

Generative adversarial networks (GANs), which are a promising type of deep generative network, have recently drawn considerable attention and made impressive progress. However, GAN models suffer from the well-known problem of mode collapse. This study focuses on this challenge and introduces a new model design, called the encoded multi-agent generative adversarial network (E-MGAN), which tackles the mode collapse problem by introducing the variational latent representations learned from a variable auto-encoder (VAE) to a multi-agent GAN. The variational latent representations are extracted from training data to replace the random noise input of the general multi-agent GANs. The generator in E-MGAN employs multiple generators and is penalized by a classifier. This integration guarantees that the proposed model not only enhances the quality of generated samples but also improves the diversity of generated samples to avoid the mode collapse problem. Moreover, extensive experiments are conducted on both a synthetic dataset and two large-scale real-world datasets. The generated samples are visualized for qualitative evaluation. The inception score (IS) and Fréchet inception distance (FID) are adopted to measure the performance of the model for quantitative assessment. The results confirmed that the proposed model achieves outstanding performances compared to other state-of-the-art GAN variants.

## 1. Introduction

Generative adversarial networks (GANs) [1], along with the rapid development of deep learning in various fields [2,3,4,5,6,7,8,9], have attracted worldwide attention in the fields of image generation [10,11], medical image analysis [12], natural language processing [13], speech emotion recognition [14,15], and others [16,17,18,19]. A GAN consists of two networks: a generator and a discriminator. The generator plays a "fraud" role by generating plausible samples from a random noise to simulate real samples, while the discriminator plays a “police” role by trying to differentiate generated fake samples from real samples [20]. These two networks compete with each other during training optimization. Thus, they form a zero-sum game that continues until Nash equilibrium is reached, at which point the generated samples are indistinguishable from real samples by the discriminator [21]. Based on this adversarial learning process, GANs can capture a complex distribution that is highly similar to the real data from a random distribution. The unique adversarial learning process causes GANs to produce sharper and more plausible samples than do other generative models. This characteristic makes GANs one of the most promising of the current deep generation models [20].

However, GAN models suffer from the well-known problem of mode collapse during the adversarial training process. With the goal of deceiving the discriminator, the generator tends to generate samples that the discriminator believes highly realistic [22]. During training, these generated samples often become limited to only a few modes rather than all the modes of the dataset, which leads to the mode collapse problem [22,23,24]. Many GAN variants have emerged to solve this problem. These variants can be divided into the following three groups: *standard GANs*, *the combination of a GAN and a VAE*, and *multi-agent GANs*.

*The standard-GAN variants* offer the most direct way to solve this problem. Among these variants, the Wasserstein GAN (WGAN) [25] is a well-known model trained by adopting the Wasserstein distance instead of the Jensen–Shannon divergence (JSD) used in typical GAN models. The Wasserstein distance is weaker than JSD but it is more continuous; thus, WGAN overcomes the problem caused by the gradient vanishing problem experienced by the JS divergence. The deep convolutional GAN (DCGAN) [26] uses the batch normalization trick to prevent mode collapse in the generator. Metz et al. proposed the unrolled GAN model [27] by defining a new objective for generator updates based on the unrolled optimization updates of the discriminator; thus, the unrolled GAN model reduces the tendency of the generator to fall into mode collapse. In [22], Salimans et al. introduced the minibatch discrimination technique. Minibatch discrimination encourages the discriminator to examine multiple generated samples in combination.

*The GAN and VAE combination* makes full use of the advantages of both a GAN and a VAE. Inspired by this idea, the Adversarial Learned Inference (ALI) [28] introduces an inference machine into a GAN to increase the number of generated sample modes. ALI learns an inverse mapping to the abstract latent feature representations from the real data and then trains on the joint distributions of data (either real or generated) and the corresponding latent variables. The mode-regularized GAN (ModeGAN) [23] encourages sample diversity by training the generator jointly with an encoder. The encoder in MDGAN serves as a regularizer that provides additional penalizing information to revise the training objectives, thereby improving the training stability and alleviating the mode collapse problem. Conditional VAE-GAN (CVAE-GAN) [29] prevents the problem via mean feature matching. The VAE-GAN structure is based on a combination of a conditional VAE and a conditional GAN.

*Multi-agent GAN variants* offer more recent promising approaches for solving the mode collapse problem. These approaches increase the diversity of the generated samples to overcome the problem by training models using multiple discriminators or multiple generators. Nguyen et al. [30] proposed a dual discriminator generative adversarial network (D2GAN) that has two discriminators and a generator. It captures multiple data patterns by minimizing the Kullback–Leibler divergence and the reverse KL divergence between the generated samples and the real data distributions using two discriminators. The generative multi-adversarial network (GMAN) [31] extends GAN models to have multiple discriminators, making this model robust to mode collapse. The mixture density generative adversarial network (MD-GAN) [24] adjusts the discriminator output using a *d*-dimensional embedding space to improve the mode discovery. Similarly to D2GAN, Ghosh et al. [32] proposed a multi-agent GAN named the message passing multi-agent generative adversarial network (MPM GAN), which tries to explore the generated sample modes more thoroughly based on message-passing between two generators. The multi-agent diverse generative adversarial network (MAD-GAN) [10] and mixture GAN (MGAN) [33] were used in an adversarial learning process using multiple generators and one discriminator to encourage generated sample diversity.

Despite their advances, these three groups of GAN variants each have some shortcomings. Variants based on standard GANs perform well on tiny or narrow-domain datasets (such as a mixture of Gaussians datasets, MNIST, LSUN), but the generated samples they learn have an incorrect anatomy on more diverse datasets (such as CIFAR-10 and STL-10) [22,27]. Variants based on a GAN and VAE combination generate high-quality images and—except for CVAE-GAN—solving the mode collapse problem is only incidental to these models [23,28]. CVAE-GAN uses a supervised training method with fine-grained category labels [29]. Currently, it is more promising to train a GAN with multi-agent architecture, because multi-agent GAN architectures increase the diversity of the generated samples by breaking through the constraints imposed by single generator-discriminator networks [33]. However, the existing multi-agent GAN models simply generate samples from a random prior distribution without exploiting the latent information contained in the real data.

**Figure 1:**
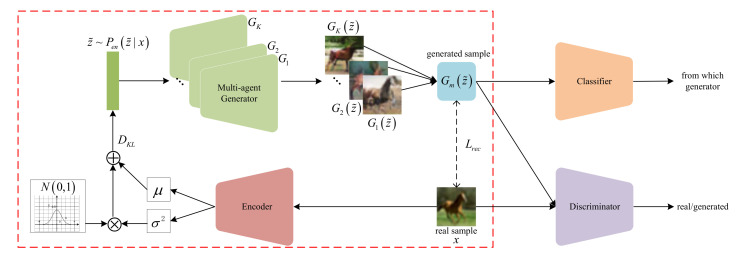
An illustration of the E-MGAN’s architecture.

In this paper, a novel GAN framework is proposed, named the encoded multi-agent generative adversarial network (E-MGAN), which aims to generate higher quality and more diverse samples by introducing the variational latent representations learned from a VAE training (as indicated by the red box in Figure 1) to a multi-agent GAN. As shown in Figure 1, the proposed E-MGAN consists of four modules: (1) an encoder, *E* (red module), which abstracts latent feature representations $\tilde{z}$ from real samples x; (2) a multi-agent generator, $G_{m}$ (green module), containing multiple generators that generate samples in different modes; (3) a classifier network, *C* (orange module), which assigns penalties with the goal of forcing the generator to discover scattered data modes; (4) and a discriminator, *D* (purple module), which distinguishes generated fake samples from real samples to maintain the adversarial learning process. Unlike the existing GAN variants, the proposed model not only makes good use of the latent feature representation condensed from real data but also uses a multi-agent generator during training to reconstruct synthetic samples in distinct modes.

Experiments are conducted on both a synthetic dataset (a 2D mixture of 25 Gaussian distributions) and two diverse, large-scale, real-world datasets (CIFAR-10 [34] and STL-10 [35]). The results are evaluated by two widely used metrics, inception scores (IS) [22] and Fréchet inception distance (FID) [36]. Note that the proposed model is an unsupervised learning method that does not require any labeled data; thus, all the results discussed in this paper are learned in an unsupervised manner. Nevertheless, the proposed model outperforms other state-of-the-art GAN variants. A detailed analysis of the diversity and realism of the generated samples is shown in Section 4. A large number of experiments demonstrate that the proposed model not only overcomes the model collapse problem but also improves the quality of the generated samples.

The key contributions of this paper are as follows:

- A novel GAN architecture is proposed, named E-MGAN, which makes good use of the advantages of both a VAE and a multi-agent GAN. The model capitalizes on the variational latent feature representations learned by VAE from real data to improve the quality of the generated samples.
- The proposed E-MGAN model surmounts the mode collapse problem by incorporating a new multi-agent generator that coordinates training with the encoder and classifier. Its input is the latent variational feature representation learned by the encoder, and its output is constrained by the classifier through maximizing the Shannon entropy. Therefore, the multi-agent generator is encouraged to generate samples in discrete data modes.
- We conducted experiments to validate the effectiveness of our model on a synthetic dataset (a 2D mixture of 25 Gaussian distributions) and two diverse, real-world datasets (CIFAR-10, and STL-10). The results illustrate that the proposed model not only overcomes the problem of model collapse, but also improves the anatomical structure of samples generated on large-scale diverse datasets.

The remainder of this paper is organized as follows. Section 2 introduces three preliminary models related to the proposed model. An in-depth study of the proposed model is provided in Section 3. Section 4 demonstrates the performance of the proposed model through a series of experiments. Finally, Section 5 provides a conclusion and briefly suggests the future work.

## 2. Preliminaries

This section provides the background regarding the proposed E-MGAN. Since the proposed model attempts to generate higher quality and more diverse samples by fully capitalizing on the advantages of a VAE and a multi-agent GAN, there related models, the original GAN, VAE, and a typical MGAN, are briefly introduced.

### 2.1. Generative Adversarial Networks (GANs)

A GAN consists of two networks that act as players in a game: a generator *G* and a discriminator *D*. *G* produces fake samples G(z) from a noise vector z, which is sampled from a prior distribution P(z). The generated fake samples G(z) imitate the real samples $x∼P_{real}\left(x\right)$ by maximizing the output of the discriminator D(G(z)). The output of the discriminator, which is denoted by D(x)∈[0,1], is the probability that the input samples x come from the real sample distribution $P_{real}\left(x\right)$; however, its input samples could also be generated samples G(z). The notation D(x)=1 means that the input sample x is a real sample. In contrast, D(x)=0 means that the discriminator will view the input sample x as a fake sample. The task of the discriminator *D* is to distinguish generated fake samples G(z) from real samples x by minimizing the probability of fake samples (denoted as D(G(z))) while maximizing that of real samples (denoted as D(x)). In this adversarial training process, the value function of GAN can be described as follows:

(1) $$V\left(G,D\right)=E_{x∼P_{real}\left(x\right)}\left[\log D(x)\right]+E_{z∼P(z)}\left[\log\left(1-D(G(z))\right)\right],$$

where $P_{real}\left(x\right)$ and P(z) are the real data distribution and a random prior distribution, respectively. From the above analysis, the training objective of GAN is a minimax game, $\min_{G}\max_{D}V\left(G,D\right)$. The parameters of the generator and those of the discriminator are alternately updated through the minimax game until the discriminator can no longer distinguish whether an input sample x or G(z) comes from the real data distribution or is a fake sample. Mathematically, this can be denoted as

$$P_{ge}\left(G\left(z\right)\right)=P_{real}\left(x\right),D\left(G\left(z\right)\right)=D\left(x\right)=\frac{1}{2},$$

where $P_{ge}\left(G\left(z\right)\right)$ is the distribution of generated samples. At this point, the GAN reaches Nash equilibrium, V(G,D)=−2log2.

### 2.2. Variational Auto-Encoder (VAE)

A VAE consists of two members: an encoder network En and a decoder network De. The encoder network En(x) compresses training data samples x into latent feature representation vectors $\tilde{z}$ with a distribution $P_{en}\left(\tilde{z}|x\right)$. Thus, the decoder network $De(\tilde{z})$ reconstructs synthetic samples $x^{\prime }∼P_{de}\left(x^{\prime }|\tilde{z}\right)$ from the abstracted latent representation vectors $\tilde{z}$. Mathematical explanations of those two networks are:

$$\begin{array}{cc} En(x) & =P_{en}\left(\tilde{z}|x\right),x∼P_{real}\left(x\right),\\ De(\tilde{z}) & =P_{de}\left(x^{\prime }|\tilde{z}\right),\tilde{z}∼P_{en}\left(\tilde{z}|x\right). \end{array}$$

The latent feature representation $P_{en}\left(\tilde{z}|x\right)$ learned from the encoder is constrained by the prior distribution P(z)∼N(0,1). $P_{en}\left(\tilde{z}|x\right)$ is the reconstructed sample distribution. Then, the training objective of VAE is to maximize its variational lower bound or evidence lower bound (ELBO) function:

(2) $$\begin{array}{cc} L_{VAE}\left(x\right)= & -D_{KL}(P_{en}\left(\tilde{z}|x\right)||P\left(z\right))+E_{\tilde{z}∼P_{en}\left(\tilde{z}|x\right)}\log P_{de}\left(x^{\prime }|\tilde{z}\right). \end{array}$$

On the right side of Equation (2), the first term is a regular term for $\theta_{en}$ that encourages the approximate posterior distribution $P_{en}\left(\tilde{z}|x\right)$ to be close to the prior distribution P(z), where $D_{KL}\left(\cdot ||\cdot \right)$ is the KL divergence. The second term is a reconstruction error. It is the maximum likelihood of the sample $x^{\prime }∼De\left(\tilde{z}\right)$ reconstructed from the extracted features $\tilde{z}∼P_{en}\left(\tilde{z}|x\right)$.

### 2.3. Mixture Generative Adversarial Nets (MGAN)

MGAN is one typical multi-agent GAN that employs multiple generators to enhance mode recovery. Assuming that there are *K* generators, each generator $G_{k}$ maps the prior z to a generated sample $x^{\prime }=G_{k}\left(z\right)$, which represents a generated distribution. Then, *K* generators can generate a mixed distribution covering *K* generated data distributions, denoted as $P_{ge}$. An MGAN includes another new member, the classifier *C*. $C_{k}\left(x^{\prime }\right)$ represents which generator $G_{k}$ the fake sample $x^{\prime }$ comes from. Therefore, MGAN is a game composed of three members: a set of generators $G_{1:K}\left(z\right)$, a discriminator D(x), and a classifier $C_{1:K}\left(x^{\prime }\right)$. The value function of MGAN is formulated as follows:

(3) $$\begin{array}{cc} & V\left(G_{1:K},C,D\right)=E_{x∼p_{real}\left(x\right)}\left[\log D(x)\right]+E_{x^{\prime }∼P_{ge}\left(z\right)}\left[\log\left(1-D\left(x^{\prime }\right)\right)\right]-\beta \sum_{k=1}^{K}E_{x^{\prime }∼P_{ge}\left(z\right)}[\log C_{k}\left(x^{\prime }\right)], \end{array}$$

where β is the diversity hyperparameter which is positive. MGAN training is a maximin game, $\min_{G_{1:K},C}\max_{D}V\left(G_{1:K},C,D\right)$.

## 3. Proposed Encoded Multi-Agent GAN

This section introduces the proposed model, encoded multi-agent GAN (E-MGAN). Differently from the existing multi-agent GAN architectures, the proposed model contains a new member, an encoder network *E*, that abstracts variational latent feature representations from real data for the generator. As shown in Figure 1, the proposed model has four members: (1) an encoder network; (2) a multi-agent generator network; (3) a classifier network; and (4) a discriminator network.

As indicated by the red box in the E-MGAN structure shown in Figure 1, the combination of the encoder network *E* and the multi-agent generator $G_{m}$ is similar to a VAE, when the multi-agent generator is considered as a decoder. First, the encoder *E* extracts the variational latent representation distribution $N(\mu ,\sigma^{2})$ from the real samples x and provides this distribution to the generator. Then, the multi-agent generator $G_{m}$ generates a fake sample $G_{m}\left(\tilde{z}\right)$ from the variational latent representation $\tilde{z}$ sampled from the posterior distribution $N(\mu ,\sigma^{2})$. However, unlike the decoder in VAE, our multi-agent generator $G_{m}$ consists of *K* generators; thus, a generated fake sample $G_{m}\left(\tilde{z}\right)$ is combined with *K* generated samples from *K* generators. After that, the generated sample $G_{m}\left(\tilde{z}\right)$ is put into the classifier *C* that recognizes which generator the fake sample comes from. The classifier encourages the generator to discover different data modes by maximizing the information entropy. Finally, the function of the multi-agent generator $G_{m}$ and the discriminator *D* is the same as that in a standard GAN. The generator $G_{m}$ tries to capture the data distribution through the gradients of discriminator *D*, while the discriminator *D* tries to distinguish the generated fake samples $G_{m}\left(\tilde{z}\right)$ from real samples x.

The remainder of this section presents the details of the proposed model in two parts. First, the formulation of the model is introduced in Section 3.1. Second, the training objective for the proposed model and the algorithm of the training process are provided in Section 3.2. Table 1 provides the notation and corresponding definitions used in the proposed model.

### 3.1. Formulation of E-MGAN

Differently from the existing multi-agent GANs, the proposed model samples from the latent feature distribution rather than from a random distribution. Recent work [37] argues that the encoder *E* of VAE can extract latent feature representations from the real data, while the extracted latent feature representations capture the semantic attributes of the training samples. Thus, E-MGAN makes good use of the advantage of a VAE that learns the variational latent feature representations from real data to improve the multi-agent GAN.

Our encoder and the selected generator form a VAE, as shown in the red box of Figure 1. The encoder *E* learns latent feature variables, the mean μ, and the covariance $\sigma^{2}$, from real data x. The posterior distribution $P_{en}\left(\tilde{z}|x\right)=N\left(\mu ,\sigma^{2}\right)$ is obtained through the reparameterization trick: $\tilde{z}=\mu +\sigma ⊙z$, where z∼N(0,1). Then, the multi-agent generator $G_{m}$ generates fake samples $x^{\prime }=G_{m}\left(\tilde{z}\right)$ from the latent feature representations $\tilde{z}∼P_{en}\left(\tilde{z}|x\right)$.

As a special VAE variant, we adopt the KL divergence and the reconstruction error in the objective function of VAE to train our encoder *E* and multi-agent $G_{m}$. The KL divergence aims to encourage the extracted feature distribution $N(\mu ,\sigma^{2})$ to be close to the random prior distribution P(z). We set the random prior distribution P(z) to a Gaussian distribution N(0,1). The analysis of the KL divergence is shown in Equation (4). The reconstruction error is used to make the reconstructed image $x^{\prime }$ more similar to the real image x. We calculate the reconstruction error $L_{rec}$ with the mean squared error (MSE) [38]. Therefore, the KL divergence and the reconstruction error in our model are as follows:

(4) $$\begin{array}{cc} L_{KL}= & D_{KL}\left(P_{en}\left(\tilde{z}|x\right)\|P\left(z\right)\right)=E_{\tilde{z}∼P_{en}\left(\tilde{z}|x\right)}\left[\log\frac{P_{en}\left(\tilde{z}|x\right)}{P(z)}\right]\\ = & E_{\tilde{z}∼P_{en}\left(\tilde{z}|x\right)}\left[\log P_{en}\left(\tilde{z}|x\right)\right]-E_{\tilde{z}∼P_{en}\left(\tilde{z}|x\right)}\left[\log P(z)\right]\\ = & -H\left(P_{en}\left(\tilde{z}|x\right)\right)+H\left(P_{en}\left(\tilde{z}|x\right),P(z)\right)\\ = & \frac{1}{2}\left[\mu^{T}\mu +sum\left(\sigma^{2}-\log\sigma^{2}-1\right)\right], \end{array}$$

(5) $$\begin{array}{cc} L_{rec}= & -E_{\tilde{z}∼P_{en}\left(\tilde{z}|x\right)}\left[\log P_{ge}\left(\tilde{z}\right)\right]≃\frac{1}{2}{∥x^{\prime }-x∥}_{2}^{2}.\;\; \end{array}$$

The last equality of Equation (4) holds, because of P(z)=N(0,1).

**Figure 2:**
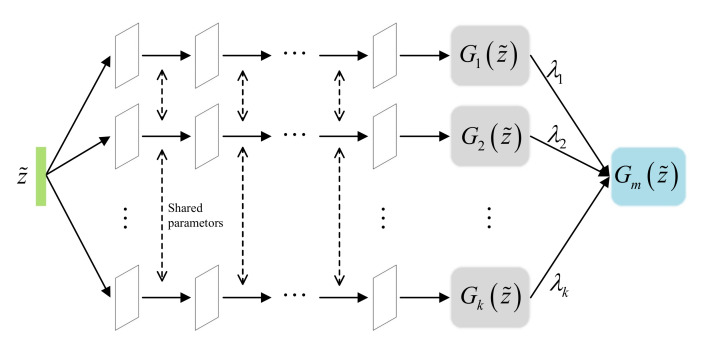
An inside view of the multi-agent generator $G_{m}$.

The multi-agent generator $G_{m}$ consists of *K* generators, as shown in Figure 2, which is inspired from MGAN [33]. However, they are different; our multi-agent generator $G_{m}$ samples from the latent feature distribution $P_{en}\left(\tilde{z}|x\right)$ learned by the encoder *E*, while the MGAN generator samples from a random prior distribution P(z). This change not only encourages our generator to capture the data distribution more quickly but also helps it generate more realistic samples that can fool the discriminator. As described in Figure 2, the generators in the multi-agent generator share their parameters except for the input layer and output layer, which helps reduce redundant computations while ensuring that the generated samples of each generator differ. Assuming that each generator $G_{i}$ captures one mode $P_{G_{i}}$ from the learned latent feature representations $\tilde{z}$, then the multi-agent generator $G_{m}$ can theoretically capture *K* data modes, named $P_{ge}\left(\tilde{z}\right)$. However, it cannot guarantee that those data modes will overlap with each other.

The classifier *C* is a penalty to the generator, encouraging the generator to discover diverse data modes. It calculates the probability ($C_{G_{i}}\left(x^{\prime }\right)$) that the input artificial samples $x^{\prime }$ come from the generator $G_{i}$. We denote the output of our generator as $G_{m}\left(\tilde{z}\right)=\sum_{i=1}^{K}\lambda_{i}G_{i}\left(\tilde{z}\right)$, where $G_{i}\left(\tilde{z}\right)∼P_{G_{i}}\left(\tilde{z}\right)$ is the output of the *i*-th generator and $\lambda_{i}$ is the weight of $G_{i}\left(\tilde{z}\right)$. Therefore, maximizing the information entropy of the classifier output is organized as the objective function of the classifier $L_{C}$:

(6) $$\begin{array}{cc} L_{C}= & \sum_{i=1}^{K}\lambda_{i}E_{x^{\prime }∼P_{G_{i}}}\left[logC_{G_{i}}\left(x^{\prime }\right)\right]=\sum_{i=1}^{K}\lambda_{i}E_{x^{\prime }∼P_{G_{i}}}\left[\log\frac{\lambda_{i}P_{G_{i}}}{\sum_{i=1}^{K}\lambda_{i}P_{G_{i}}}\right]\\ = & \sum_{i=1}^{K}\lambda_{i}E_{x^{\prime }∼P_{G_{i}}}\left[\log\frac{P_{G_{i}}}{\sum_{i=1}^{K}\lambda_{i}P_{G_{i}}}\right]+E_{\lambda_{i}}\log\lambda_{i}\\ = & \sum_{i=1}^{K}\lambda_{i}E_{x^{\prime }∼P_{G_{i}}}\left[\log P_{G_{i}}\right]-\sum_{i=1}^{K}\lambda_{i}E_{x^{\prime }∼P_{G_{i}}}\left[\log\sum_{i=1}^{K}\lambda_{i}P_{G_{i}}\right]+E_{\lambda_{i}}\log\lambda_{i}\\ = & -\sum_{i=1}^{K}\lambda_{i}H\left(P_{G_{i}}\right)+H\left(\sum_{i=1}^{K}\lambda_{i}P_{G_{i}}\right)+H\left(\lambda_{i}\right)\\ = & JSD\left(P_{G_{1}},\ldots ,P_{G_{m}}\right)+H\left(\lambda_{i}\right). \end{array}$$

The Jensen-Shannon divergence (JSD) of multiple distributions that $JSD\left(P_{1},\ldots ,P_{m}\right)=H\left(\sum_{i=1}^{K}\lambda_{i}P_{i}\right)-\sum_{i=1}^{K}\lambda_{i}H\left(P_{i}\right)$ is given in [39]. According to the above analysis, maximizing $L_{C}$ implies maximizing the JSD among $P_{G_{1}},P_{G_{2}},\cdots ,P_{G_{K}}$ and the information entropy of $\lambda_{i}$. The greater the JSD is, the more dispersed the generated sample modes are. Therefore, maximizing $L_{C}$ encourages our multi-agent generator to generate samples in relatively dispersed data modes. $H(\lambda_{i})$ reaches its maximum value when $\lambda_{i}=1/K$; thus, we set $\lambda_{i}=1/K$ in our model. Clearly, the classifier functions as a penalty term to the generator.

The role of our discriminator *D* is the same as that in a classical GAN; its task is to distinguish the generated samples $G_{m}\left(\tilde{z}\right)$ from real samples x. It calculates the probability D(x)∈[0,1] that an input sample x (a real sample x or a generated sample $x^{\prime }$) is sampled from the real data distribution $P_{real}\left(x\right)$. It accomplishes this task by maximizing the probability of real samples while reducing the probability of generated samples. Therefore, the objective function of the discriminator is to maximize the following function:

(7) $$\begin{array}{cc} L_{D}= & E_{x∼P_{real}}\left[\log D(x)\right]+E_{x^{\prime }∼P_{ge}}\left[\log(1-D\left(x^{\prime }\right)\right]. \end{array}$$

This process motivates the generator to generate more realistic samples with the latent feature representation, thereby improving its ability to generate samples.

According to the above analysis, the goal of our multi-agent generator $G_{m}$ is not only to generate more realistic samples that fool the discriminator but also to improve the mode recovery of generated samples to avoid the mode collapse problem. First, our multi-agent generator $G_{m}$ samples from the latent feature representation $\tilde{z}∼P_{en}\left(\tilde{z}|x\right)$ provided by the encoder. Second, its generated samples $x^{\prime }=G_{m}\left(\tilde{z}\right)$ are penalized by the reconstruction error $L_{rec}$ and the value function of the classifier Lc. Finally, the generated samples $x^{\prime }$ must maximize the probability $D(x^{\prime })$ to fool the discriminator. Therefore, the training objective function of the multi-agent generator is described as minimizing the value function $L_{G_{m}}$:

(8) $$\begin{array}{cc} L_{G_{m}}= & E_{x^{\prime }∼P_{ge}}\left[\log(1-D\left(x^{\prime }\right)\right]+L_{rec}-L_{C}. \end{array}$$

### 3.2. Objective of E-MGAN

The latent representation distribution $P_{en}\left(\tilde{z}|x\right)$ extracted by the proposed model from real samples x needs to minimize the KL distance $L_{KL}$. The proposed model samples from the learned distribution $P_{en}\left(\tilde{z}|x\right)$ to generate samples. To encourage the generated samples $x^{\prime }$ to be more plausible and realist samples, they are subject to reconstruction error $L_{rec}$ by minimizing Equation (5). The generated samples aim to deceive the discriminator by minimizing $L_{D}$. Simultaneously, the discriminator tells the generated samples from real samples by maximizing $L_{D}$. Therefore, the total training objective of E-MGAN can be summarized as follows:

(9) $$\begin{array}{cc} \min_{E,G_{m},C}\max_{D}V\left(E,G_{m},C,D\right)= & E_{x∼P_{real}}\left[\log D(x)\right]+E_{x^{\prime }∼P_{ge}}\left[\log(1-D\left(x^{\prime }\right)\right]+L_{KL}+L_{rec}-L_{C}\\ = & E_{x∼P_{real}}\left[\log D(x)\right]+E_{x^{\prime }∼P_{ge}}\left[\log(1-D\left(x^{\prime }\right)\right]\\ & +D_{KL}\left(P_{en}\left(\tilde{z}|x\right)\|P\left(z\right)\right)-\frac{1}{2}{∥x^{\prime }-x∥}_{2}^{2}-\sum_{i=1}^{K}\lambda_{i}E_{x^{\prime }∼P_{G_{i}}}\left[\log C\left(x^{\prime }\right)\right], \end{array}$$

where the calculation formulas for the 3rd and 4th terms in Equation (9) are shown by Equation (4) and Equation (5), respectively.

Algorithm 1 describes the training process of the proposed E-MGAN model. First, the *K* generators in the multi-agent generator, $\lambda_{i}$ (i=0,1,…,K), are initialized with the weights of the *i*th generator; and the parameters of the encoder network, multi-agent generator network, classifier network, and discriminator network are initialized with $\theta_{E}$, $\theta_{G}$, $\theta_{G}$, and $\theta_{D}$. Then, the encoder extracts the latent feature distribution at Step 4. The two parts of encoder loss $L_{E}$, the KL divergence $D_{KL}\left(P_{en}\left(\tilde{z}|x\right)\|P\left(z\right)\right)$ between the learned latent feature distribution $P_{en}\left(\tilde{z}|x\right)$ and the random prior distribution P(z), and the reconstruction error $L_{rec}$, are calculated at Step 5 and Step 8, respectively. Thus, the encoder loss $L_{E}$ is obtained in Step 9. In addition, the loss functions of the classifier, generator, and discriminator are calculated at Steps 11, 13, and 14, respectively. Finally, Steps 15–18 update the parameters of the four networks.

**Algorithm 1** The training process of the proposed E-MGAN algorithm.
**Require:***K*, the number of generators in multi-agent generator; $\lambda_{i}$, the *i*-th generator weight; $P_{real}\left(x\right)$, the real data distribution; P(z)∼N(0,1), the random prior distribution.
1:**Initialize:**$\theta_{E},\theta_{G},\theta_{G},$ and $\theta_{D}$, the parameters of the encoder network, generator network, classifier network and discriminator network, respectively.2:**while** not converged **do**3:  Sample $x∼P_{real}\left(x\right)$ a batch from the real dataset;4:  $P_{en}\left(\tilde{z}|x\right)\leftarrow Enc(x);$5:  $L_{KL}\leftarrow D_{KL}\left(P_{en}\left(\tilde{z}|x\right)\|P\left(z\right)\right);$6:  Sample a minibatch of $\tilde{z}∼P_{en}\left(\tilde{z}|x\right);$ put into multi-agent generator;7:  $x^{\prime }\leftarrow G_{m}\left(\tilde{z}\right);$8:  $L_{rec}\leftarrow {∥x^{\prime }-x∥}_{2}^{2};$9:  $L_{E}\leftarrow L_{KL}+L_{rec};$10:  Put a minibatch of reconstruction samples $x^{\prime }$ into the classifier and obtain $C(x^{\prime });$11:  $L_{C}\leftarrow -\sum_{i=1}^{K}\lambda_{i}E_{x^{\prime }∼P_{G_{i}}}\left[logC_{G_{i}}\left(x^{\prime }\right)\right];$12:  Put a minibatch of training samples x and reconstruction samples $x^{\prime }$ the discriminator and obtain D(x) and $D(x^{\prime });$13:  $L_{G}\leftarrow \log\left(1-D\left(x^{\prime }\right)\right)+L_{KL}+L_{rec}-L_{C};$14:  $L_{D}\leftarrow -(\log\left(D\left(x\right)\right)+\log(1-D\left(x^{\prime }\right));$15:  $\theta_{E}\overset{+}{⟵}-\nabla_{\theta_{E}}(L_{E});$16:  $\theta_{G}\overset{+}{⟵}-\nabla_{\theta_{G}}(L_{G});$17:  $\theta_{D}\overset{+}{⟵}-\nabla_{\theta_{D}}(L_{D});$18:  $\theta_{C}\overset{+}{⟵}-\nabla_{\theta_{C}}(L_{C});$19:
**end while**



## 4. Experiments

In this section, comprehensive experiments on both a synthetic dataset and two real-world datasets are described; they are done to validate the performance of the proposed E-MGAN model. The generated samples are shown for evaluation via visual inspection for qualitative assessment. Meanwhile, for quantitative assessment, the generated samples are evaluated by two of the currently most widely adopted metrics, namely, IS and FID. The experimental details and results are reported in the remainder of this section.

### 4.1. Datasets

A 2D synthetic dataset and two widely used large-scale real-world datasets, CIFAR-10 [34] and STL-10 [35] are adopted to demonstrate the performance of the proposed model through a series of experiments.

**Figure 3:**
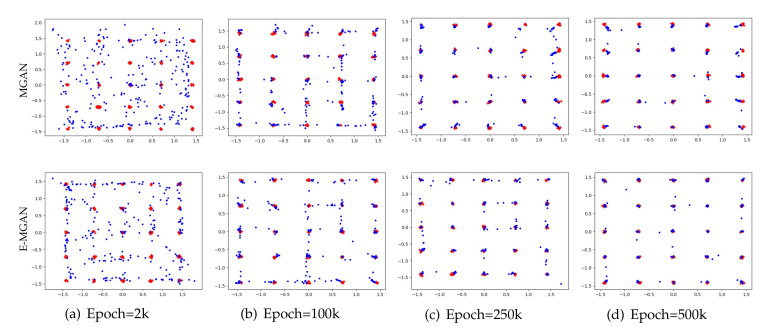
Samples generated by MGAN (in the upper row) and our proposed E-MGAN (in the bottom row) trained on the 2D synthetic dataset. The red points are real samples, while the blue points are generated samples.

- **2D synthetic dataset** adopted in these experiments consisted of 25 isotropic Gaussian distributions with a fixed standard deviation of 0.05. These 25 Gaussian distributions are arranged in a 5×5 grid, as shown by the red points in Figure 3.
- **CIFAE-10** [34] contains 60,000 32×32 color natural images and was collected by Alex Krizhevsky, Vinod Nair, and Geoffrey Hinton. These images are balanced across the following 10 categories: airplane, automobile, bird, cat, deer, dog, frog, horse, ship, and truck. There are 6000 images in each category.
- **STL-10** [35] consists of 100,000 unlabeled natural color images balanced across the following 10 categories: airplane, car, bird, cat, dog, deer, horse, monkey, ship, and struck. STL-10 is more diverse than the CIFAR-10 dataset, and it contains images with a resolution of 96×96. To ensure a fair comparison with other models, we compressed the images from 96×96 resolution to 48×48 resolution.

### 4.2. Evaluation Metrics

The evaluation of generated images is still a notoriously challenging issue, and there is no uniform standard. Among current metrics, IS [22] and FID [36] are the two most widely adopted evaluation metrics in various GAN variants. In this paper, for quantitative assessment, these two different and widely used metrics are adopted to estimate the quality and diversity of generated samples in the experiments.

- **Inception score (IS)** [22] is widely used to measure sample qualities. It is wonderfully designed and based on Google’s inception deep learning model. The IS is adopted to evaluate the results in the experiments because it assesses the generated images based on two image aspects: realism and diversity. The score is computed as follows:
  (10) $$IS=\exp\left(E_{x^{\prime }∼P_{ge}}D_{KL}(P\left(y|x^{\prime }\right)||P\left(y\right))\right).$$
  As shown in Equation (10), IS consists of two parts: p(y|x) and p(y). The former is a conditional label distribution for each given generated image $x^{\prime }$. Lower entropy means the generated sample $x^{\prime }$ is closer to a certain category that contains meaningful objects. The latter $p\left(y\right)=\int_{x}p(y|x)dx$, a marginal distribution, is the label distribution of all generated samples. Higher entropy implies the generated samples are scattered among different categories. In these experiments, the IS is adopted to evaluate the realism and diversity of generated samples using the code available from https://github.com/openai/improved-gan/tree/master/inception_score.
- **Fréchet inception distance (FID)** [36] is another reasonable way to quantify the quality of generated images. FID is more consistent than IS because it evaluates the generated samples by calculating the Fréchet distance between the real samples and generated samples in the feature space, where the Fréchet distance is a Wasserstein-2 distance. Therefore, FID is better than IS at capturing the level of similarity between the generated samples and real samples. If the feature distribution of real samples and that of generated samples are respectively denoted as $N(\mu_{r},\Sigma_{r})$ and $N(\mu_{g},\Sigma_{g})$, the FID value between them can be calculated by
  $$\begin{array}{cc} FID= & d^{2}\left(N\left(\mu_{r},\Sigma_{r}\right),N\left(\mu_{g},\Sigma_{g}\right)\right)\\ = & ∥\mu_{r}-\mu_{g}{∥}_{2}^{2}+Tr\left(\Sigma_{r}+\Sigma_{g}-2{\left(\Sigma_{r}\Sigma_{g}\right)}^{\frac{1}{2}}\right). \end{array}$$
  In addition, FID is proven sensitive to mode dropping, particularly in respect to intraclass mode dropping [40]. We supplemented the experiments with FID to evaluate the diversity of generated data modes. The pertinent code is abstracted from https://github.com/bioinf-jku/TTUR.

### 4.3. Experimental Settings

To ensure a fair comparison, we followed the experimental settings of previous works. All the experiments are implemented using Python 3.7 and TensorFlow [41] with two GT2080Ti GPUs and CUDA 10.0.

There are four networks in the proposed model. First, the encoder network *E* is designed by adopting a multi-layer perceptron, a simple neural network that extracts data characteristics. Second, as described in Figure 2, the multi-agent generator consists of *K* generators that shares all their parameters except for the input and output layers. Therefore, in fact, they share the same hidden layer of a single network except for their input and output layers. In addition, for fair comparison with previous works, we adopted DCGAN [42] to construct both our multi-agent generator $G_{m}$ and our discriminator *D* networks, allowing us to use the same network architecture and training procedure. DCGAN is a widely adopted modeling architecture that has been used in various GAN variants due to its stability during adversarial training using convolutional neural networks. Finally, because the classifier *C* and the discriminator *D* are essentially both classifiers, to reduce redundant calculations, they are designed to share parameters except in the last output layer, as shown in Figure 1. The difference between them is that the latter is a binary classifier, while the former is a multi-class classifier that calculates the probability that a particular generator generated a given sample; the goal is to identify which generator was the originator of each synthetic sample.

### 4.4. Experimental Results

The experiments are divided into two parts to validate the proposed model. On one hand, the experiments demonstrate that our model improves the quality of the generated samples with respect to the precision of synthetic data and the correct anatomy of real images. On the other hand, the experiments demonstrate that the proposed model effectively improves the diversity of the generated samples. The samples generated by the proposed model are more diverse not only among the categories but also within the categories. It is worth noting that our model requires no data labels. The proposed model is a completely unsupervised method. Therefore, all the results are generated in an unsupervised manner.

#### 4.4.1. Quality Analysis of the Generated Samples

#### Results on Synthetic Samples

A Gaussian mixture distribution with 25 isotropic data modes is selected to validate the accuracy of the proposed model. The proposed E-MGAN model is compared with a typical multi-agent GAN, MGAN. The experimental results are shown in Figure 3. Each model is trained for 50,000 epochs and the results of the different methods are presented (MGAN in the upper row and E-MGAN in the bottom row) at different epochs: 2k, 5k, 25k, and 50k.

As shown in Figure 3, both E-MGAN and MGAN [33] capture all 25 modes. However, E-MGAN converges much faster than does MGAN; it covers nearly all the modes as early as step 2k, while the generated points of MGAN display more freedom. Furthermore, E-MGAN captures the data modes more precisely than does MGAN from Epoch 25k to the end, while there are several modes that MGAN cannot exactly cover. This comparison between MGAN and E-MGAN on the 2D synthetic dataset demonstrates that E-MGAN captures data modes more accurately.

#### Results on Real-World Samples

**Figure 4:**
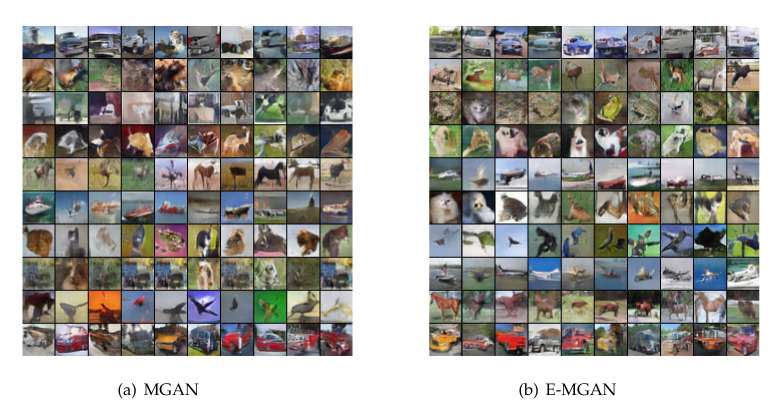
Generated samples of proposed E-MGAN and MGAN trained on CIFAE-10.

Figure 4 shows the samples generated by the proposed E-MGAN and those generated by MGAN trained on the CIFAR-10 dataset. We adopted 10 generators to train these two multi-agent GAN models and present the generated samples of different generators in the rows, as shown in Figure 4. The samples generated by MGAN are displayed in Figure 4a, while the samples generated by E-MGAN are displayed in Figure 4b. In the left-hand image, MGAN can identify horses, ships, cars or trucks, flying objects (birds or airplanes), and other unclear animals. However, there is clearly a generator collapse in MGAN. In the right-hand image, E-MGAN clearly identifies the outlines of horses, ships, birds, and automobiles. It can also roughly identify the cats and dogs, trucks, and even frogs. More importantly, no generator suffers from the mode collapse problem, although the deer could not be clearly identified.

To quantitatively analyze the quality of the generated samples, Table 2 shows the IS results of samples generated by E-MGAN and other benchmark methods on both the CIFAR-10 and STL-10 datasets. It also lists the IS results of the real data, which act as the baseline for all the generated samples from the different models. The models compared on the CIFAR-10 dataset include several classic GAN variants (such as WGAN [25], MIX+WGAN [43], improved-GAN [22], DCGAN [26], and MAGAN [44]); models that combine a GAN and VAE (such as ALI [28] and BEGAN [45]); and several multi-agent GANs (such as GMAN [31], D2GAN [30], and MGAN [33]). Among the compared models, we chose the best performing MGAN for recovery. We obtained an IS of 8.12±0.10 for MGAN through 500 epochs of 5000 samples in the tested implementation, which is slightly lower than the results published in [33], as shown in Table 2. By adopting 10 generators as in MGAN, we completed the experiment with the proposed E-MGAN model. The samples generated by E-MGAN achieved a slightly higher IS of 8.42±0.09, which is closest to the real data, indicating that E-MGAN outperforms other models.

Table 2 also presents the IS results of E-MGAN compared with other models on STL-10. The STL-10 dataset is more diverse than CIFAR-10 because it is a subset of the full ImageNet dataset. The compared models include DCGAN [26], D2GAN [30], and MGAN [33]. The images in the STL-10 dataset have a resolution of 96×96; however, DCGAN, D2GAN, and MGAN are all trained on images with a resolution of 48×48. To ensure a fair comparison with these previous works, we compressed the images of the STL-10 dataset from 96×96 to 48×48. We reproduced the experimental results of the MGAN model on the STL-10 dataset and obtained an IS score of 9.12±0.10, which is somewhat lower than the experimental result of 9.22 reported in [33]. In the same experimental environment, the proposed model obtained an IS score of 9.35±0.10, outperforming the other baselines (DCGAN and D2GAN). Similarly, on the STL-10 dataset, the E-MGAN model achieved an IS value as high as 9.35±0.12. Obtaining the highest IS score indicates that the quality of samples generated by the proposed E-MGAN model is superior to that of the other models.

#### 4.4.2. Diversity Analysis of the Generated Samples

#### Results on Synthetic Samples

**Figure 5:**
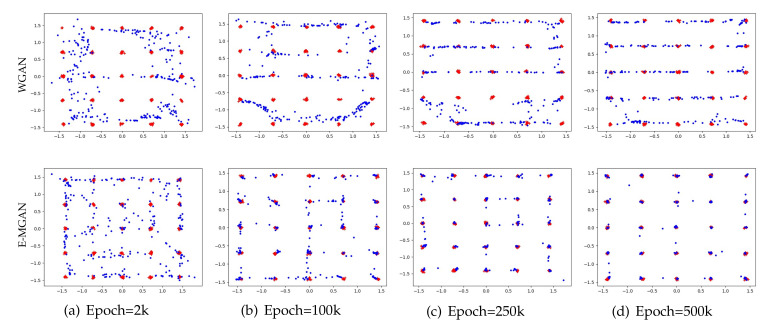
Samples generated by WGAN (in the upper row) and our proposed E-MGAN (in the bottom row) trained on the 2D synthetic dataset. The red points are real samples, while the blue points are generated samples.

Figure 5 compares the experimental results of E-MGAN and WGAN trained on 25 independent Gaussian mixture distribution datasets (WGAN is in the upper row and E-MGAN is in the lower row). E-MGAN converges much faster than WGAN because it achieves an even distribution around all the targets (red points) and nearly covers most of the targets as early as epoch 2k. By epoch 10k, E-MGAN recognizes all modes. However, at epoch 2k, the points generated by WGAN are distributed around merely a few target modes, and multiple target modes in the middle are not found. Until the end, at epoch 50k, several targets are still not discovered (such as col 1, row 5; col 3, row 5; col 4, row 2 and 3; and col 5, row 4). Additionally, during the entire WGAN training process, most of the generated points wander between two adjacent target modes. The wandering points among the points generated by E-MGAN gradually decrease and gather around the target points. Finally, at epoch 50k, only a few points are floating between two adjacent target points. Experiments on the synthetic dataset of WGAN and E-MGAN proved that the proposed E-MGAN model generates more diverse data to discover all modes and thus overcomes the mode collapse problem.

#### Results on Real-World Samples

**Figure 6:**
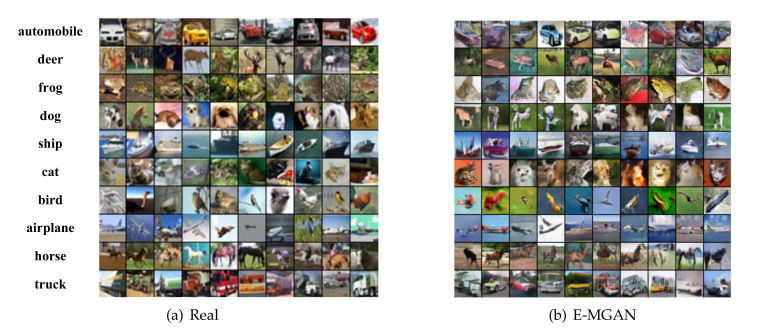
Samples on the CIFAR-10 dataset. On the left are real data sampled from the CIFAR-10 dataset, and on the right are samples generated by E-MGAN trained on CIFAR-10 dataset.

**Figure 7:**
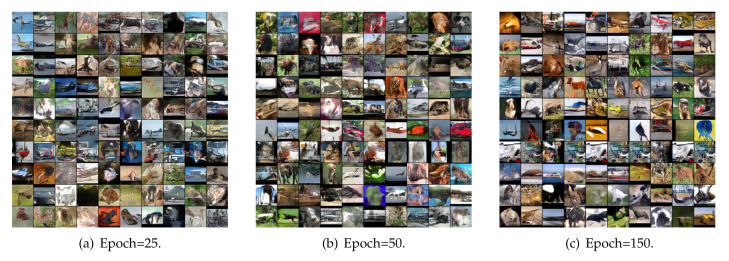
Samples generated by the proposed E-MGAN at different epochs trained on STL-10.

To qualitatively illustrate the performance of the proposed model regarding the diversity of the generated samples, we intuitively evaluated the image quality by exhibiting the generated samples trained on different real-word datasets. Figure 6 presents images generated by our model trained on the CIFAR-10 32×32 dataset. Some generated samples trained on the STL-10 48×48 dataset are shown in Figure 7.

Figure 6 shows the real samples (in Figure 6a) and the generated samples of the E-MGAN model (in Figure 6b) on the CIFAR-10 dataset. Figure 6a shows 10 examples of each category among the 10 categories of the real dataset. Figure 6b shows 10 samples of each of the categories generated by the E-MGAN model. On one hand, the generated samples of E-MGAN cover all modes; cars, deer, frogs, dogs, ships, birds, airplanes, horses, and trucks can be clearly identified. The cats are fuzzy because the samples in the real dataset are insufficient to be clearly identified. On the other hand, the generated samples in each category are different. The generated samples of cars, ships, dogs, horses, and trucks are viewed from different perspectives and appear in diverse colors. The generated data on the CIFAR-10 dataset demonstrate that E-MGAN achieves diversity both among classes and within classes.

Figure 7 represents generated samples of the proposed E-MGAN model trained on STL-10 at different epochs. Recognizing all the objects in the generated images on STL-10 is somewhat difficult, but it is relatively easy to recognize the shapes of objects such as cars, trucks, ships, airplanes, birds, horses, and other animals. Furthermore, the colors and backgrounds (such as the sky, sea, and land) of the generated images become increasingly diverse as the number of training iterations increases.

To quantitatively evaluate the performance of the E-MGAN model regarding diversity, FID is a good measurement method [40]. The FID values are compared with previous works, including DCGAN [26], DCGAN + TTUR [36], WGAN-GP, WGAN-GP-TTUR [36], and MGAN, on the CIFAR-10 dataset to validate the ability of the proposed model to solve the mode collapse problem. The FID values of E-MGAN and MGAN are obtained through experiments and the FID results of other models are quoted as reported in [36]. As shown in Table 3, the proposed E-MGAN obtained the lowest FID value (24.4) among all models. Obtaining the lowest FID value means that E-MGAN is the model least affected by the mode collapse problem and that it can recover different modes. The samples generated by the proposed E-MGAN model are shown in Figure 6b.

## 5. Conclusions

This paper proposes a novel multi-agent GAN architecture E-MGAN. Differently from existing multi-agent GANs, the proposed model aims to generate higher quality and more diverse samples by making full use of the advantages of a VAE and a multi-agent GAN. The E-MGAN learns the variational latent representations from real data to improve the quality of the generated samples. Meanwhile, E-MGAN increases the diversity of generated samples via the coordinated training of a multi-agent generator with the encoder, the classifier, and the discriminator. Extensive experiments on both a synthetic dataset and two large-scale real-world datasets, (CIFAR-10 and STL-10), demonstrated that the proposed E-MGAN model not only improves the quality of the generated samples but also recovers diverse data modes. Future work should consider employing labeled data information or utilizing the statistical information of generated samples to increase the diversity of generative models.

## Figures and Tables

**Table 1:** Notation used in the model of E-MGAN.

Notation	Definition	Notation	Definition
x	Real samples	$P_{real}\left(x\right)$	Real data distribution
z	Random prior variable	P(z)	Random prior distribution
$\mu ,\sigma^{2}$	Mean and variance of latent feature representations	$P_{en}\left(\tilde{z}|x\right)$	Latent feature distribution
$\tilde{z}$	Latent feature representations	$G_{m}\left(\tilde{z}\right)$	Output of the multi-agent generator
*K*	Number of generators in multi-agent generator.	$D_{KL}\left(\cdot ||\cdot \right)$	Kullback–Leibler (KL) divergence
$L_{KL}$	Loss of Kullback–Leibler (KL) divergence	H(·)	Shannon entropy
$L_{rec}$	Reconstruction error	H(·,·)	Cross–entropy
$x^{\prime }$	Reconstructed (generated) samples	sum(·)	Sum function of vector elements
$P_{G_{i}}$	Generated data mode of *i*-th generator	$P_{ge}$	Generated sample distribution
$\lambda_{i}$	Weight of $G_{i}\left(\tilde{z}\right)$	$C_{G_{i}}\left(x^{\prime }\right)$	Probability that $x^{\prime }$ comes from $G_{i}$
$L_{C}$	Value function of the classifier	JSD(·)	Jensen–Shannon divergence
$L_{D}$	Value function of the discriminator	D(x)	Probability that x is a real sample
$L_{G_{m}}$	Value function of the multi-agent generator		

**Table 2:** Inception scores on CIFAR-10 and STL-10 datasets. All the results are made in an unsupervised manner. The higher the IS value, the better the quality of generated samples is. A dash (“–”) indicates unavailable data.

Model	CIFAR-10	STL-10
Real data	11.24±0.16	26.08±0.26
WGAN [25]	3.82±0.06	–
MIX+WGAN [43]	4.04±0.07	–
mproved-GAN [22]	4.36±0.04	–
ALI [28]	5.34±0.05	–
BEGAN [45]	5.62	–
MAGAN [44]	6.40±0.03	–
GMAN [31]	6.00±0.19	–
DCGAN [26]	6.40±0.05	7.54
DFM [46]	7.72±0.13	8.51±0.13
D2GAN [30]	7.15±0.07	7.98
MGAN [33]	8.33±0.10	9.22±0.11
**E-MGAN**	8.42±0.13	9.35±0.12

**Table 3:** FIDs of different models on CIFAR-10. The lower the FID value, the better the diversity of generated samples is.

Model	FID
DCGAN [26]	37.7
DCGAN + TTUR [36]	36.9
WGAN-GP [47]	29.3
WGAN-GP + TTUR [36]	24.8
MGAN [33]	26.7
**E-MGAN**	24.4
